# Phosphonic acid-containing inhibitors of tyrosyl-DNA phosphodiesterase 1

**DOI:** 10.3389/fchem.2022.910953

**Published:** 2022-08-16

**Authors:** Xue Zhi Zhao, Wenjie Wang, George T. Lountos, Joseph E. Tropea, Danielle Needle, Yves Pommier, Terrence R. Burke

**Affiliations:** ^1^ Chemical Biology Laboratory, Center for Cancer Research, National Cancer Institute, National Institutes of Health, Frederick, MD, United States; ^2^ Developmental Therapeutics Branch and Laboratory of Molecular Pharmacology, Center for Cancer Research, National Cancer Institute, National Institutes of Health, Bethesda, MD, United States; ^3^ Basic Science Program, Frederick National Laboratory for Cancer Research, Frederick, MD, United States; ^4^ Center for Structural Biology, Center for Cancer Research, National Cancer Institute, Frederick, MD, United States

**Keywords:** tyrosyl-DNA phosphodiesterase 1, phosphonic acid, 3′-processed substrate, one-pot Groebke-Blackburn-Bienayme multicomponent reactions, imidazopyrazines, imidazopyridines

## Abstract

Tyrosyl-DNA phosphodiesterase 1 (TDP1) repairs stalled type I topoisomerase (TOP1)-DNA complexes by hydrolyzing the phosphodiester bond between the TOP1 Y723 residue and the 3′-phosphate of its DNA substrate. Although TDP1 antagonists could potentially reduce the dose of TOP1 inhibitors needed to achieve effective anticancer effects, the development of validated TDP1 inhibitors has proven to be challenging. This may, in part, be due to the open and extended nature of the TOP1 substrate binding region. We have previously reported imidazopyrazines and imidazopyridines that can inhibit TDP1 catalytic function *in vitro*. We solved the TDP1 crystal structures with bound inhibitors of this class and found that the dicarboxylic acid functionality within the *N*-(3,4-dicarboxyphenyl)-2-diphenylimidazo [*1,2-a*]pyridin-3-amine platform overlaps with aspects of phosphoryl substrate recognition. Yet phosphonic acids could potentially better-replicate cognate TOP1-DNA substrate binding interactions than carboxylic acids. As reported herein, we designed phosphonic acid-containing variants of our previously reported carboxylic acid-containing imidazopyrazine and imidazopyridine inhibitors and effected their synthesis using one-pot Groebke–Blackburn–Bienayme multicomponent reactions. We obtained crystal structures of TDP1 complexed with a subset of inhibitors. We discuss binding interactions of these inhibitors within the context of phosphate-containing substrate and carboxylic acid-based inhibitors. These compounds represent a new structural class of small molecule ligands that mimic aspects of the 3′-processed substrate that results from TDP1 catalysis.

## Graphical Abstract

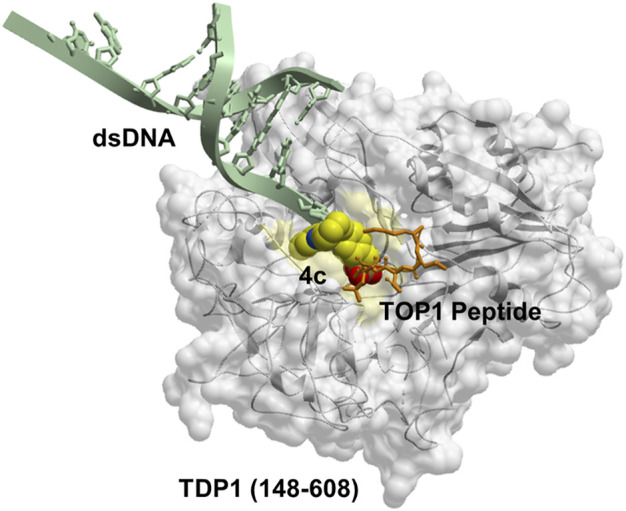

## Introduction

**FIGURE 1:**
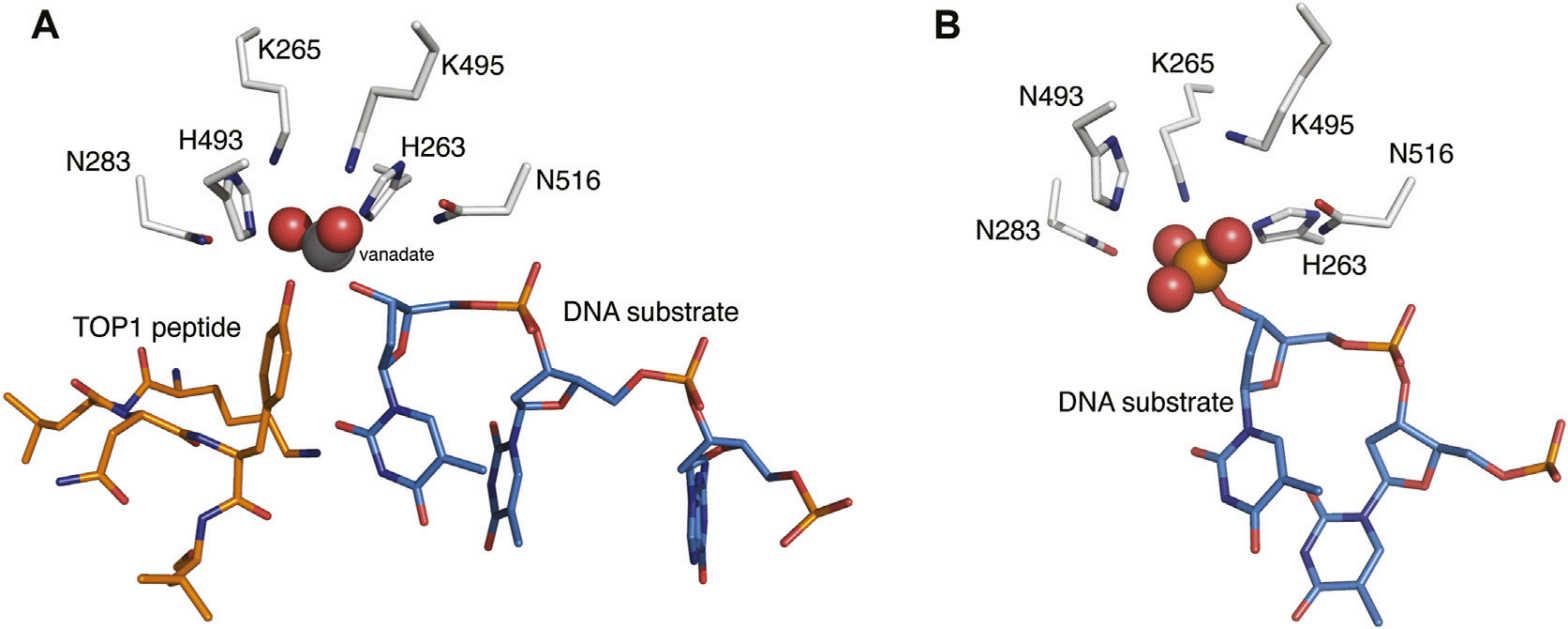
The binding modes of TDP1 substrates. **(A)** Structure of TDP1 (148–608) (carbon atoms in gray, nitrogen atoms in blue, oxygen atoms in red) complexed to single DNA strand (carbon atoms in light blue) and TOP1 peptide (carbon atoms in orange) with the phosphate mimetic vanadate highlighted in a sphere representation (PDB code: 1NOP (Davies et al., 2003)). **(B)** Structure of TDP1 complexed to a DNA strand (carbon atoms in light blue) with the phosphonic acid highlighted in a sphere representation (PDB code: 5NWA (Flett et al., 2018)).

Human tyrosyl-DNA phosphodiesterase 1 (TDP1) is a 608 amino acid DNA repair enzyme that processes 3′-DNA end-blocking lesions by hydrolyzing the 3′-phosphate esters. TDP1 is of importance because it can reverse stalled type I topoisomerase (TOP1)-DNA cleavage complexes (TOP1cc′s) by cleaving the phosphodiester bond between the TOP1 Y723 residue and the 3′-phosphate of its DNA substrate (Pommier, 2006; 2009). TDP1 inhibitors could potentiate the anticancer activity of TOP1 inhibitors (Pommier et al., 2014; Hu et al., 2016; Laev et al., 2016) and as such, they would represent a new therapeutic class that could potentially be used for the treatment of cancer in combination with current TOP1 inhibitors (Interthal et al., 2005; Beretta et al., 2010; Comeaux and van Waardenburg, 2014; Gao et al., 2014). When acting on TOP1cc′s, the target phosphate ester bond nestles in a well-formed pocket that contains the signature “His-Lys-Asn” (HKN) catalytic residues (Figure 1A) (Interthal et al., 2001; Davies et al., 2004; Raymond et al., 2004). The DNA 3′-phosphate ester bond is hydrolyzed directly without a requirement for cofactors or metal ions (Yang et al., 1996; Davies et al., 2003, 2004; Nitiss, 2009; Huang et al., 2011; Ashour et al., 2015; Laev et al., 2016). A conserved H263 residue initiates an initial nucleophilic attack on the substrate tyrosyl phosphate ester. The resulting covalent enzyme–DNA complex undergoes a second nucleophilic by a water molecule under activation by H493 to release the DNA and restore the catalytic site (Davies et al., 2002).

**FIGURE 2:**
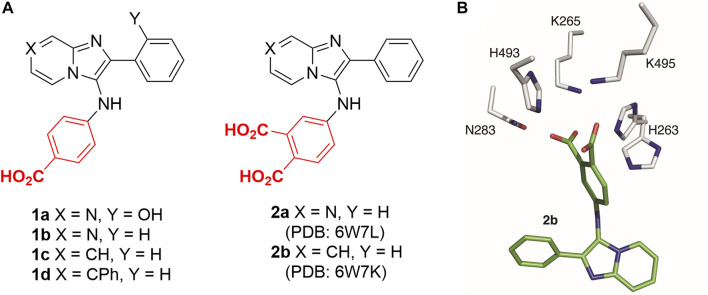
Structures of TDP1 inhibitors and the binding mode. **(A)** Structures of imidazopyrazine and imidazopyridine-based TDP1 inhibitors. **(B)** Structure of TDP1 active site with highlighted catalytic residues (gray) complexed to the TDP1 inhibitor imidazopyridine **2b** (carbon atoms in green; PDB code: 6W7K (Zhao et al., 2021)).

Although numerous small molecule TDP1 inhibitors have been reported (Dyrkheeva et al., 2021; Salomatina et al., 2021; Zhang et al., 2021), the structural basis of their interactions with the enzyme are poorly understood due to a lack of X-ray crystal structures of TDP1 bound to inhibitors. None-the-less, molecular recognition of DNA-containing substrates has been informed by crystal structures of TDP1 with vanadate or tungstate phosphate mimetics bound at the TDP1 catalytic site, as well as with DNA or substrate surrogates (Figure 1A) (Davies et al., 2003; 2004). Crystal structures of TDP1 have also been reported in complex with double-strand DNA (Figure 1B) (Flett et al., 2018). We recently undertook library-based screens to discover small molecule motifs that can bind to the TDP1 catalytic pocket. Our first crystallographic fragment screen examined more than 600 low molecular weight fragments for their ability to bind to TDP1 (Lountos et al., 2019). Most of the fragments for which crystal structures could be obtained, represent variations of hydroxyquinoline carboxylic acid or phthalic acid-containing motifs. More recently, we employed an Alexa Fluor 647 (AF647)-tagged TDP1 (148–608) fluorescent probe to determine its ability to bind to members of a small molecule microarray (SMMs) containing 21,000 drug-like small molecules. In this fashion, we identified the *N,2*-diphenylimidazo [*1,2-a*]pyrazin-3-amine nucleus and its imidazopyridine derivatives (**1a—1d**) as new TDP1-binding motifs (Figure 2A) (Zhao et al., 2021). Although we attempted to obtain crystal structures of several SMM-derived inhibitors bound to TDP1, we only succeeded with analogs (**2a** and **2b**) containing the *N*-3,4-dicarboxyphenyl motif. This indicated the importance of the binding interactions provided by the two carboxylic acids. The crystal structures reveal that the molecules of **2a** and **2b** bind to the TDP1 catalytic site in a similar mode. They can form hydrogen bonds with the catalytic HKN residues (H263, K265, N283, H493, K495 and N516) and S399 (Figure 2B). We also observed that stabilizing hydrophobic interactions with **2b** are provided by the side chain atoms of Y204, P461, and W590. These structures indicate that the ligand dicarboxylic acid functionality overlaps with aspects of phosphoryl substrate recognition as shown by previously reported crystal structures (Flett et al., 2018).

Carboxylic acids have been shown to be good phosphate bioisosteres (Burke and Lee, 2003; Elliott et al., 2012). Yet phosphonic acids could potentially better-replicate cognate TOP1-DNA substrate binding interactions than carboxylic acids. As we report herein, we have designed phosphonic acid-containing variants of our previously reported carboxylic acid-containing imidazopyrazine and imidazopyridine inhibitors and we have obtained crystal structures of the catalytic domain of TDP1 (148–608) complexed with a subset of these inhibitors. We discuss the binding interactions of these inhibitors within the context of phosphate-containing substrate and carboxylic acid-based inhibitors.

## Results and discussion

### Design and synthesis of phosphonic acid-containing analogs

**FIGURE 3:**
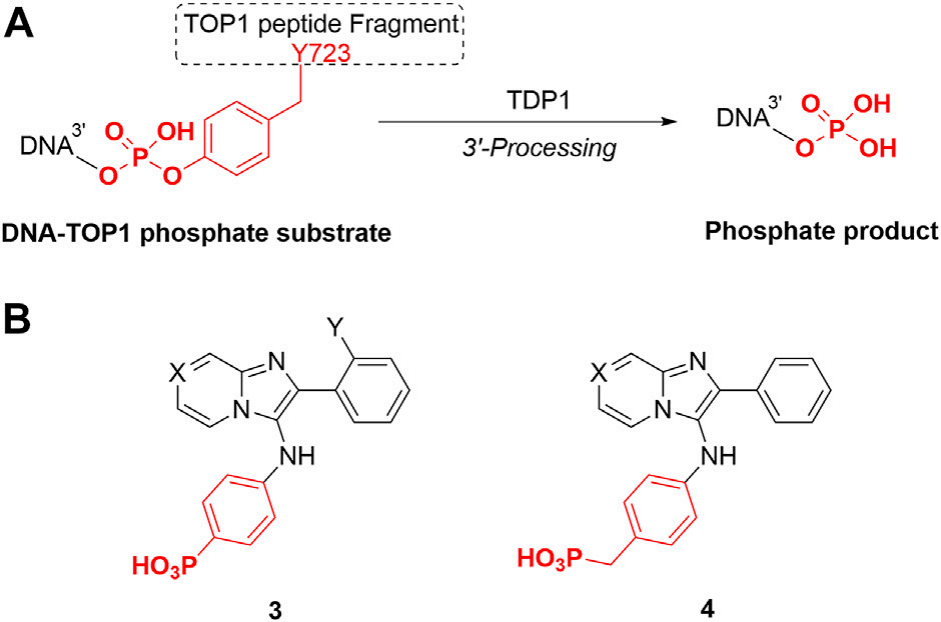
TDP1 hydrolysis reaction and phosphonic acid-containing TDP1 inhibitors. **(A)** TDP1 hydrolyzes the phosphotyrosyl between the residue Y723 of TOP1 peptide fragment and the 3′-end of the DNA substrate to generate a 3′-processed product. **(B)** General structures of phenylphosphonic acid **3** and benzylphosphonic acid **4** to mimic phosphotyrosyl substrate and 3′-processed phosphate product.

TDP1 hydrolyzes the DNA-TOP1 phosphate substrate by cleaving the 3′-phosphoryl ester to form the 3′-processed phosphate product (Figure 3A). Benzylphosphonic acid and phenylphosphonic acids can be effective phosphate replacements within the context of phosphotyrosyl (pTyr)-dependent signal transduction inhibitors (Burke and Lee, 2003). These pharmacophores have also shown utility in polynucleotide-utilizing enzymes, such as in replacement of the ribose-5-phosphate moiety in eukaryotic initiation factor 4E (eIF4E)-binding guanosine-5-monophosphate with 8-substitued benzyl and phenylphosphonic acid and carboxyphenyl-containing guanine groups (Chen et al., 2012). We designed phenylphosphonic acids **3** and benzylphosphonic acids **4** (Figure 3B) to examine the effects of replacing mono-carboxylic and dicarboxylic acid functionality in the SMM-derived inhibitors **1a—1d** and **2a** and **2b**, respectively. We prepared these analogs using Groebke-Blackburn-Bienayme multicomponent one-pot reactions (GBBR) employing readily available aldehyde, pyridin-2-amine and isocyanide building blocks (Bienayme and Bouzid, 1998; Blackburn et al., 1998; Groebke et al., 1998; Shaaban and Abdel-Wahab, 2016).

**SCHEME 1:**
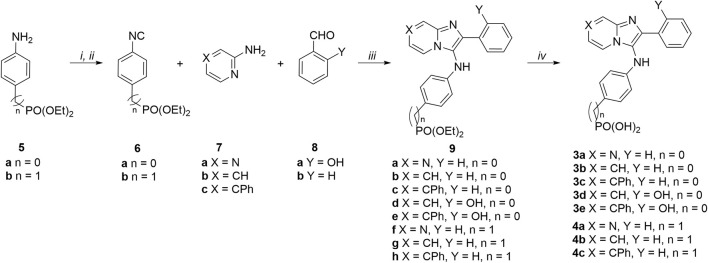
Synthesis of phosphonic acid-containing analogues **3a—3e** and **4a—4c**. Reagents and conditions: (i) HCO_2_H, Ac_2_O, 55°C then amines (**5a** and **5b**), THF, 0°C; (ii) POCl_3_, Et_3_N, THF, 0°C; (iii) pyrazine-2-amine (**7a)** or pyridine-2-amines (**7b** and **7c**)_,_ aldehydes (**8a** and **8b**), and isonitriles (**6a** and **6b**), CH_3_CO_2_H, MeOH, rt; (iv) TMSBr, CH_3_CN, rt.

Phenylphosphonic acids (**3a—3e)** and benzylphosphonic acids (**4a—4c)** were synthesized starting from diethyl (4-isocyanophenyl)phosphonate (**6a**) and diethyl (4-isocyanobenzyl)phosphonate (**6b**) (Scheme 1). These key compounds were prepared from commercially available diethyl (4-aminophenyl)phosphonate (**5a**) and diethyl (4-aminobenzyl)phosphonate (**5b**) in two steps as we previously reported (Zhao et al., 2021). Amines **5a** and **5b** were reacted with freshly prepared acetic formic anhydride (from formic acid and acetic anhydride). The resulting formamidobenzenes partially isomerized to the corresponding phenylformimidic acids (complex NMR peaks due to formamide rotamers). The mixtures of formamidobenzene and isomeric phenylformimidic acid were treated with phosphoryl trichloride and triethylamine in THF to provide the isonitriles **6a** and **6b**, respectively. The isonitriles were subject to one-pot GBBR multicomponent reactions with amines [pyrazin-2-amine (**7a**), pyridin-2-amine (**7b**) or 4-phenylpyridin-2-amine (**7c**)] and aldehydes [benzylaldehyde (**8a**) or 2-hydroxylbenzylaldehyde (**8b**)] to afford the diethyl phosphonate-containing compounds **9a**—**9h**. The diethyl phosphonates were deprotected using trimethylsilyl bromide (McKenna et al., 1977; Kim et al., 2001) to yield the final phenylphosphonic acids **3a—3e** and benzylphosphonic acids **4a—4c**.

## Biological evaluation

**FIGURE 4:**
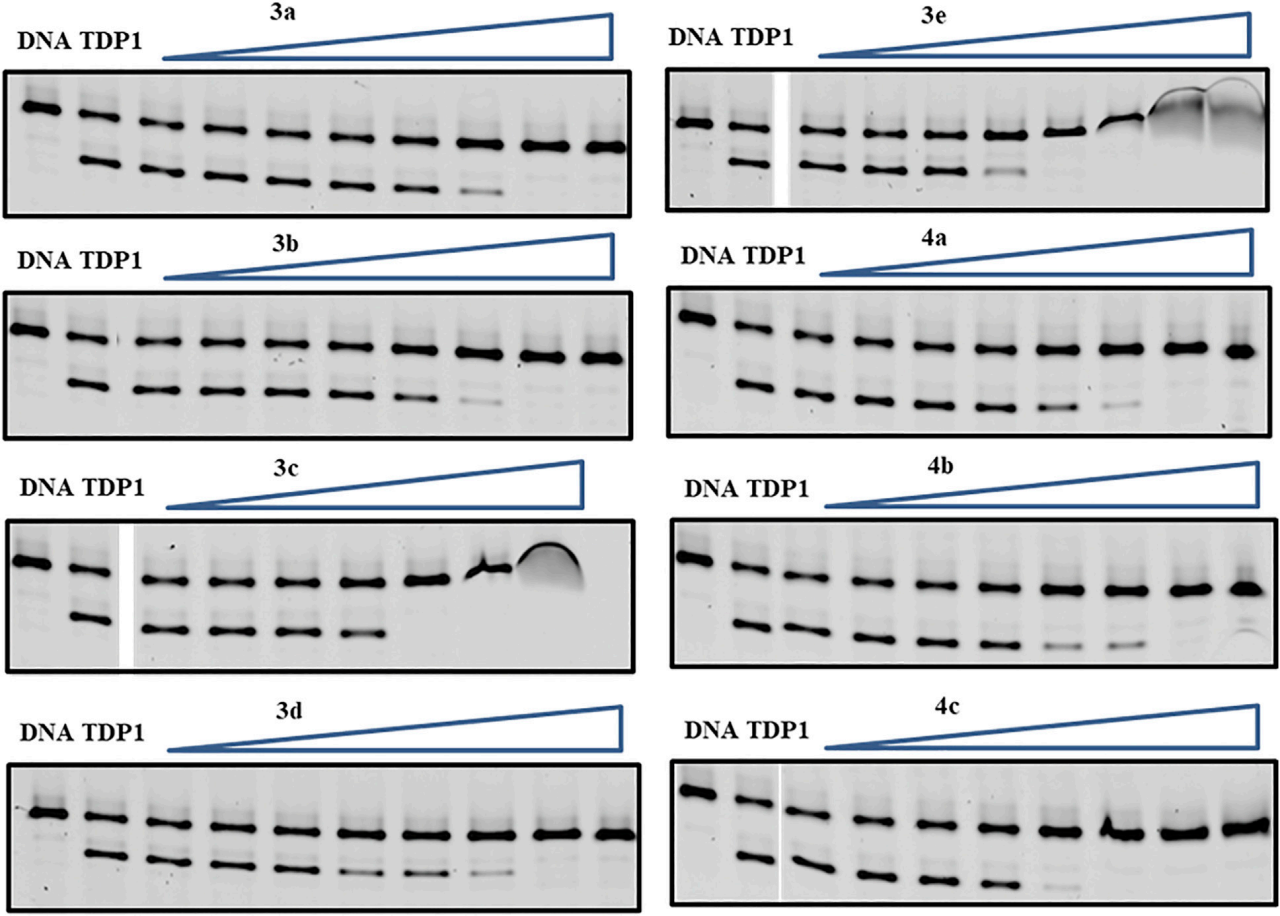
Representative gels for inhibition of full-length TDP1-catalyzed hydrolysis by phenylphosphonic acids **3a—3e** and benzylphosphonic acids **4a—4c**. In each gel: lane 1, N14Y only; lane 2, N14Y and TDP1; lanes 3–10, 3-fold serial dilution of drugs from 12 μM to 27 mM except for **3c** from 12 μM to 9 mM. The crescent vague bands in **3c** and **3e** are caused by the poor drug solubility in these high concentrations.

The synthetic products were examined for their ability to inhibit TDP1 catalysis in *in vitro* gel-based fluorescence assays as previously described (Lountos et al., 2019; Zhao et al., 2021). A 5′-Cy5-labelled DNA substrate (N14Y, 5′-Cy5-GATCTAAAAGACTT-pY-3′) was incubated in TDP1 reaction buffer with recombinant TDP1 (10 p.m.) or truncated TDP1 (148–608) in the absence or presence of inhibitors for 15 min at room temperature. Inhibitors were evaluated at concentrations ranging from 12 μM to 27 mM (Figure 4). The TDP1 inhibition values were calculated based on gel images of cleavage product (N14P, 5′-Cy5-GATCTAAAAGACTT-p-3′) shown in Figure 4 and listed in Table 1.

**TABLE 1:** Structures and inhibitory potencies of TDP1 inhibitors in gel-based fluorescence assay with full-length TDP1 or TDP1 (148–608) *in vitro*.

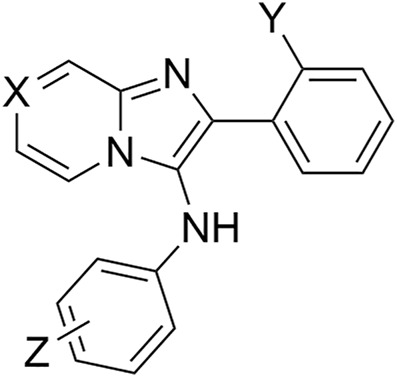					
Compound^a^	X	Y	Z	TDP1 IC_50_ (µM)^b^	TDP1 (148–608) IC_50_ (µM)^c^
**1a**	N	OH	4′-CO_2_H	19 ± 5.9	--
**1b**	N	H	4′-CO_2_H	0.71 ± 0.03	--
**1c**	CH	H	4′-CO_2_H	8.7 ± 1.8	--
**1d**	CPh	H	4′-CO_2_H	2.98 ± 0.24	--
**2a**	N	H	3′,4′-diCO_2_H	>100	--
**2b**	CH	H	3′,4′-diCO_2_H	15.9 ± 1.2	--
**3a**	N	H	4′-PO_3_H	2448 ± 36	4719 ± 430
**3b**	CH	H	4′-PO_3_H	1660 ± 160	3811 ± 751
**3c**	CPh	H	4′-PO_3_H	545 ± 8	225.2 ± 2.3
**3d**	CH	OH	4′-PO_3_H	1575 ± 166	4477 ± 50
**3e**	CPh	OH	4′-PO_3_H	292.5 ± 7.5	605 ± 32
**4a**	N	H	4′-CH_2_PO_3_H	1280 ± 112	4358 ± 472
**4b**	CH	H	4′-CH_2_PO_3_H	1650 ± 850	4813 ± 228
**4c**	CPh	H	4′-CH_2_PO_3_H	583.5 ± 11.5	1186 ± 350

a Compounds **1a—1d** and **2a** and **2b** have been reported in our previous paper (Zhao et al., 2021).

b The half maximal inhibitory concentration (IC_50_) values have been evaluated by gel-based fluorescence assay *in vitro* using full-length TDP1. Every experiment was repeated three times independently

c The half maximal inhibitory concentration (IC_50_) values have been evaluated by gel-based fluorescence assay *in vitro* using TDP1 (148–608). Every experiment was repeated three times independently

The mono-carboxylic acid-containing analogs **1a—1d** showed greater inhibition than the dicarboxylic acid-containing analogs **2a** and **2b** in gel-based fluorescence assay *in vitro* using full-length TDP1 (Table 1) (Zhao et al., 2021). The phosphonic acid-containing analogs **3a—3e** and **4a—4c** showed poorer inhibition (IC_50_ values from µM to mM) overall. However, while having the same phenylphosphonic acid headgroup, the imidazopyridine analogues (**3b**, IC_50_ = 1660 ± 160 µM and **3d**, IC_50_ = 1575 ± 166 µM) showed slightly increased TDP1 inhibitory potencies relative to the imidazopyrazine-containing analog (**3a**, IC_50_ = 2448 ± 36 µM). Adding a 7-phenyl group to the imidazopyridine analogs (**3c**, IC_50_ = 545 ± 8 μM and **3e**, IC_50_ = 292.5 ± 7.5 µM) increased inhibitory potencies from 3- to 5-fold relative to **3b** and **3d**. Adding a 7-phenyl substituent to the benzylphosphonic acid-containing analog **4b** (IC_50_ = 1650 ± 850 µM) increased inhibitory potency approximately 3-fold (**4c**, IC_50_ = 583.5 ± 11.5 µM). Overall, the phosphonic acid-containing inhibitors were less potent than the corresponding carboxylic-based inhibitors. This seems counter-intuitive, since phosphonic acids should be more high-fidelity phosphate isosteres than carboxylic acid-based moieties. Full-length TDP1 includes TDP1 catalytic domain residues 148–608 and allosteric binding domain residues 1–147 (Krumpe et al., 2020). To confirm that these phosphonic acid-containing analogs are binding at the catalytic domain, we also examined the analogs in gel-based fluorescence assay *in vitro* using truncated TDP1 (148–608) (Table 1). Without the TDP1 allosteric binding domain residues (1–147), most of the analogs (**3a**, **3b** and **3d** and **4a—4c**) show 2 to 3-fold loss of inhibitory potencies in the assay with TDP1 (148–608) as compared with the full-length TDP1. Compound **3c** shows 2-fold more potent using catalytic domain TDP1 (148–608) (IC_50_ = 225.2 ± 2.3 µM) relative to what was observed using full-length TDP1 (IC_50_ = 545 ± 8 µM). This may suggest more specific catalytic domain-binding affinity.

### X-ray crystal structures of TDP1-bound analogs

**FIGURE 5:**
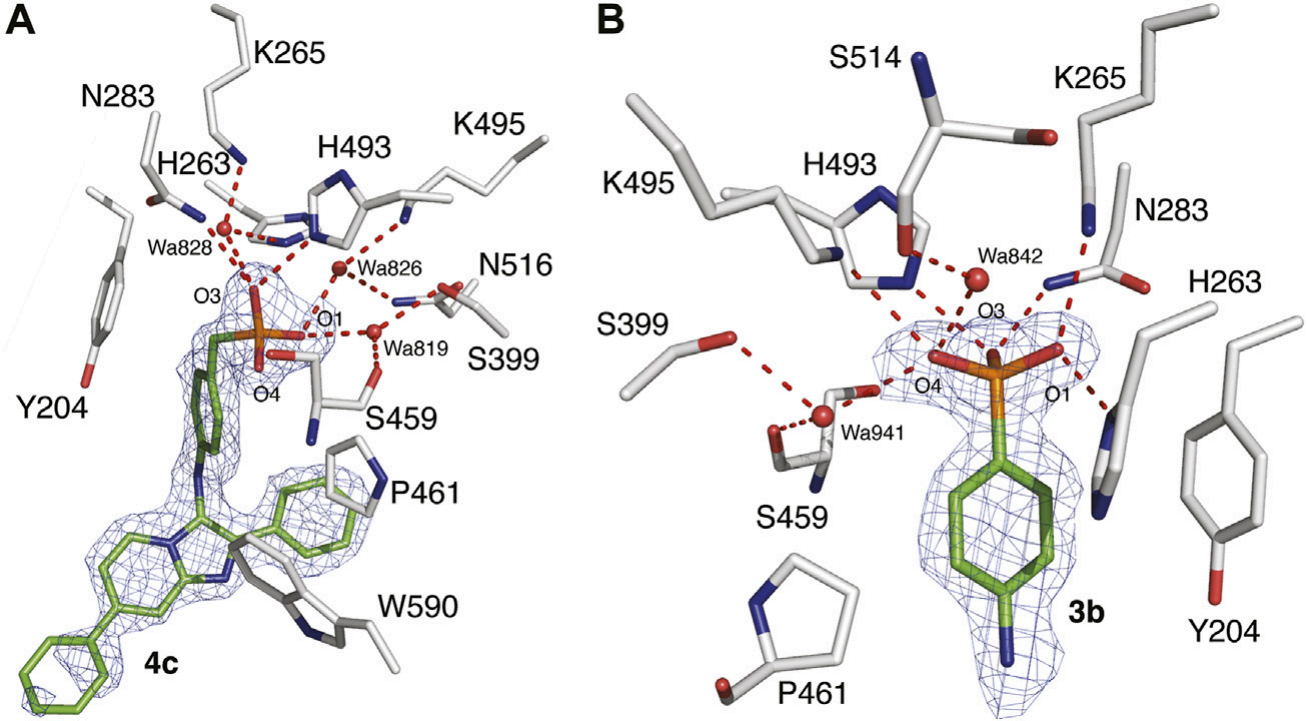
X-ray crystal structures of TDP1 bound to compounds **4c** and **3b**. **(A)** Structural representation of the TDP1 active site (chain B, carbon atoms in gray) complexed to compound **4c** (carbon atoms in green, PDB code: 7UFY). Hydrogen bonds are depicted as red dashes. The fit of **4c** to the 2*F*_*o*_ *-F*_*c*_ electron density map (blue, 1.58 Å resolution, 0.9σ level contour) is shown. **(B)** Structural representation of the TDP1 active site (chain A) complexed to compound **3b** (PDB code: 7UFZ). The fit of **3b** to the 2*F*_*o*_ *-F*_*c*_ electron density map (blue, 1.56 Å resolution, 1.0σ level contour) is shown.

We have previously reported the X-ray crystal structures of dicarboxylic acid-containing compounds **2a** and **2b** bound to the TDP1 catalytic pocket (Zhao et al., 2021). As we previously reported, compounds **2a** and **2b** bind to the active site with their carboxylate headgroups engaged to the catalytic HKN residues (H263, K265, N283, H493, K495 and N516) and S399. Stabilizing hydrophobic interactions are provided by the side chain atoms of Y204, P461, and W590. In our current work, we obtained X-ray diffraction data from crystals soaked with compounds **3b** and **4c** at 1.56 Å and 1.58 Å resolution, respectively (Figure 5 and Supplementary Table S1). Unambiguous electron density for compound **4c** was observed in chain B of TDP1 while the active site pocket of chain A had no compound bound. Compound **4c** was observed to bind to the active site of TDP1 with the benzylphosphonic acid headgroup nestled at the base of pocket lined with the catalytic residues (Figure 5A). Direct hydrogen bonding interactions are observed between the phosphonic acid 03 oxygen and the of the side chain nitrogen atoms of N283 (3.0 Å) and H493 (2.7 Å). Additional binding interactions occur via a network of water-mediated hydrogen bonds between the 03 oxygen of the phosphonic headgroup and water 828 (2.9 Å) with the side chains of K265 (2.6 Å) and H263 (3.2 Å). The 01 oxygen atom of the phosphonic acid headgroup is engaged in a water-mediated hydrogen bond network via hydrogen bonding to water 819 (2.6 Å) which is hydrogen bonded to the side chain oxygen atom of S399 (2.6 Å) and the backbone carbonyl oxygen of S459 (2.8 Å). An additional water-bridged hydrogen bonding network occurs between the 01 oxygen atom of the phosphonic acid headgroup and the side chain nitrogen atoms of K495 (2.7 Å) and N516 (3.0 Å) via water 826 (2.6 Å). The 04 oxygen atom of the phosphonic acid headgroup, however, does not hydrogen bond to any of the active site residues. The Y204 side chain stacks against the aryl moiety of compound **4c** via a hydrophobic interaction. Additional hydrophobic interactions occur between the side chain of P461 with the phenyl ring of **4c** and W590 with the imidazopyridine. Additional electron density for compound **4c** was observed in between crystal-packing monomers of TDP1. Compound **4c** was found sandwiched between residue K175 (chain A) and P593 (chain B of a crystallographic symmetry mate). The location of this molecule is likely a crystallographic artifact.

In the 1.56 Å data set collected from the TDP1 crystal soaked with **3b**, electron density for the arylphosphonic acid headgroup was observed in the active site of both monomers of TDP1 in the asymmetric unit (Figure 5B). However, there was no clear electron density observed for the rest of the molecule (imidazopyridine) indicating that this region is disordered likely due to the presence of a rotatable bond between the amino linker and the imidazopyridine moiety. Nevertheless, the well-defined electron density for the arylphosphonic acid headgroup gives important structural insights into the binding interactions to the active site pocket (Figure 5B). Two additional molecules of **3b** were also visible in the electron density maps, however, they were found to be inserted between packing interfaces of TDP1 monomers with crystallographic symmetry mates suggesting that they are likely crystallographic artifacts.

Analysis of the binding mode of the arylphosphonic headgroup demonstrates that each of the oxygen atoms of the phosphonic acid is engaged in binding interactions with the signature HKN catalytic motifs of TDP1. The 01 oxygen atom hydrogen bonds directly to the side chain nitrogen atoms of the K265 and H263 (2.8 Å). The 04 oxygen atom is hydrogen bonded to the side chain nitrogen atom of K495 (3.0 Å) and is engaged in a water-mediated hydrogen bonding network with water 842 (2.8 Å) and the backbone carbonyl oxygen of S514 (2.6 Å). An additional water-bridged interaction occurs via water 941 (3.2 Å) and the Oγ oxygen atom of the S399 side chain (2.8 Å) and the backbone carbonyl oxygen of S459 (3.0 Å). The 03 oxygen atom is hydrogen bonded to the side chain nitrogen atoms of the N283 (2.8 Å) and H493 (2.6 Å). Stabilizing hydrophobic interactions occur between the side chains of Y204 and P461 with the aryl moiety of **3b**. Additionally, the side chain of H263 is positioned such that the Cε2 carbon atom provides a hydrophobic interaction with the aryl ring of **3b**.

### Comparison of binding modes

**FIGURE 6:**
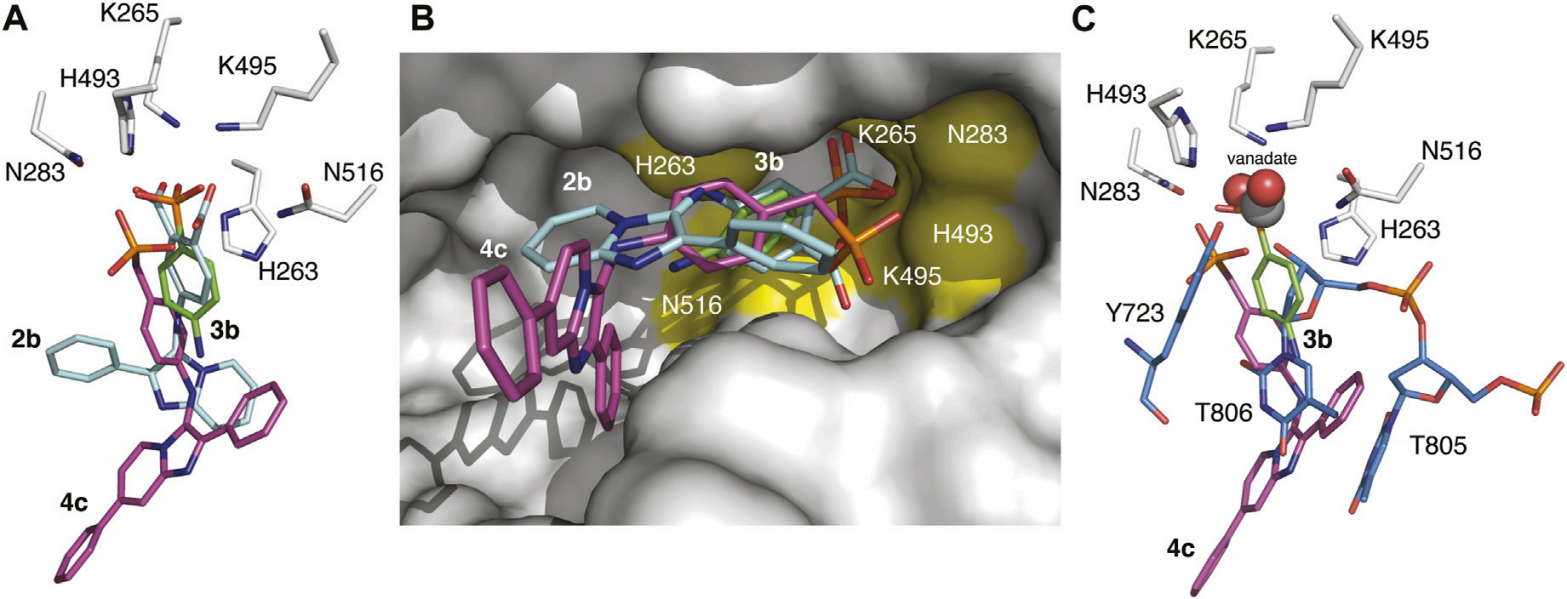
Overlaid structures of TDP1 with bound small molecules and substrate. **(A)** Active site structures of TDP1 bound to compound **2b** (carbon atoms in cyan, PDB code: 6W7K) and **3b** (carbon atoms in green, PDB code: 7UFZ) superimposed onto the structure of TDP1 bound to compound **4c** (carbon atoms in magenta, PDB code: 7UFY). **(B)** Surface representation of TDP1 (gray) showing the orientation of compounds **2b**, **3b**, and **4c** in the active site groove. The catalytic HKN residues are highlighted in yellow. **(C)** Active site structures of TDP1 bound to compound **3b** and of TDP1 bound to vanadate, ssDNA substrate, and a TOP1-derived peptide (carbon atoms in light blue, PDB code: 1NOP) superimposed onto the coordinates of TDP1 bound to compound **4c**.

The structures of TDP1 bound to **3b** and **4c** were superimposed to compare the binding modes of the compounds (Figure 6A). The extension from the phenyl ring caused by the addition of a benzylic methylene unit has a substantial impact on the binding orientation of the phosphonic acid headgroup. In compound **3b**, the phosphonic acid headgroup is positioned approximately at the center of the HKN motif while the phosphonic acid of **4c** is shifted approximately 2.6 Å (with respect to the phosphate atom) towards the H493 and K495 residues in comparison with **3b**. This results in fewer direct hydrogen bonding interactions with the catalytic residues of TDP1 but binding interactions with the catalytic residues are indeed picked up via several water-mediated bridges. It is worth noting that all oxygen atoms of the phosphonic acid in **3b** are involved in hydrogen bonding interactions with the catalytic residues but the oxygen 04 atom of the phosphonic acid in compound **4c** is not hydrogen bonded to any of the active site residues. The aryl ring of the benzylphosphonic acid headgroup is shifted approximately 2.5 Å away from where the aryl ring of **3b** is positioned and is also observed to be rotated approximately 90°. Therefore, the positional differences in the binding modes of the benzylphosphonic acid and arylphosphonic acid headgroups provides different structural space to optimize these two different classes of compounds (Figure 6B).

When compared to the binding mode of **2b**, the phosphonic acid headgroup of **3b** superimposes closely with the carboxylate group of **2b** (shift of 0.8 Å in distance) and the aryl moieties align closely with a slight rotation of the aryl moiety in **3b** (Figures 6A,B). The position of the phosphonic headgroup therefore enables interactions with the same residues that interact with the dicarboxylates. Comparing the position of **2b** and **4c**, there is a major difference in the binding modes. The shift in the binding of the benzylphosphonic acid headgroup results in a major difference in the position of the imidazopyridine moiety that imparts a very different spatial orientation of the imidazopyridine moiety in the active site groove.

The structures were also superimposed with the coordinates of a TDP1-vanadate-TOP1 peptide-DNA complex (PDB code: 1NOP) which gives us a snapshot of the transition state mimetic of the reaction (Figure 6C). Interactions of the vanadate phosphate mimetic and DNA 3′-phosphate group correspond closely to what we observed with the phosphonic acid in **3b**. However, the benzylphosphonic acid moiety in **4c** is displaced by approximately 2.9 Å with respect to the vanadate and 3′-phosphate. The phosphonic acid of **4c** superimposes closely with the hydroxyl group of the Y723 side chain from the bound TOP1-derived peptide that is covalently attached to the vanadate (which mimics the transition state). Based on analysis of these structural alignments, the arylphosphonic moiety is positioned more closely with the dicarboxylate moieties whereas the benzylphosphonic acid headgroup differs markedly giving rise to different binding modes that may provide different routes for structural optimization of the inhibitors. Nevertheless, experimental evidence of the binding of both the benzylphosphonic acid and arylphosphonic acid headgroups confirm two new varieties of TDP1 inhibitors that may possess more favorable properties for drug design over the previously identified di-carboxylate containing inhibitors. This opens opportunities for designing new classes of TDP1 inhibitors.

## Conclusion

We designed a series of phosphonic acid-containing analogues that were intended to mimic the phosphotyrosyl substrate and 3′-processed phosphate product based on imidazopyridine-containing TDP1 inhibitors from SMM leads. These analogs show micromolar to millimolar inhibitions in gel-based fluorescence assay *in vitro* using either full-length TDP1 or catalytic domain TDP1 (148–608). The phenylphosphonic acid containing analog **3c** shows more specific binding to TDP1 catalytic domain in the TDP1 fluorescence assay *in vitro*. X-ray crystal structures of the inhibitors **3b** and **4c** bound to TDP1 reveal that the phenylphosphonic acid headgroup and the benzylphosphonic acid headgroups have different binding modes compared with the bioisosteric dicarboxyphenyl motif in **2b**. A vanadate phosphate mimetic (PDB code: 1NOP) and the 3′-phosphate group in a TDP1-bound duplex DNA substrate (PDB code: 5NWA) more closely correspond to the phenylphosphonic acid in **3b**. However, the benzylphosphonic acid moiety in **4c** is displaced by approximately 2.9 Å with respect to the vanadate and 3′-phosphate. These results identify new varieties of TDP1 inhibitors that may possess more favorable properties to bind TDP1 while engaging the catalytic pocket along with the DNA substrate-binding channels. These compounds provide the first phosphonic acid-containing small molecule ligands capable of accessing the catalytic pocket of TDP1. This structural information offers new insights into binding modes of phosphate mimetics within the TDP1 catalytic site.

## Experimental section

### General synthesis

Proton (^1^H), carbon (^13^C) and phosphine (^31^P) NMR spectra were recorded on a Varian 400 MHz spectrometer or a Varian 500 MHz spectrometer and are reported in ppm relative to TMS and referenced to the solvent in which the spectra were collected. Solvent was removed by rotary evaporation under reduced pressure, and anhydrous solvents were obtained commercially and used without further drying. Purification by silica gel chromatography was performed using CombiFlash with EtOAc−hexanes solvent systems. Preparative high pressure liquid chromatography (HPLC) was conducted using a Waters Prep LC4000 system having photodiode array detection and Phenomenex C18 columns (catalogue no. 00G-4436-P0-AX, 250 mm × 21.2 mm 10 μm particle size, 110 Å pore) at a flow rate of 20 ml/min. Binary solvent systems consisting of A = 0.1% aqueous TFA and B = 0.1% TFA in acetonitrile were employed with gradients as indicated. Products were obtained as amorphous solids following lyophilization. Electrospray ionization-mass spectrometric (ESI-MS) were acquired with an Agilent LC/MSD system equipped with a multimode ion source. High resolution mass spectrometric (HRMS) were acquired by LC/MS-ESI using LTQ-Orbitrap-XL at 30K resolution.

### General procedure A for synthesis of substituted isocyanobenzenes (6a and 6b)

A mixture of formic acid (60 mmol) and acetic anhydride (66 mmol) was stirred (55°C, 2 h) and then cooled to rt to afford acetic formic anhydride. The mixture was added dropwise to a solution of substituted aminobenzene (**5a** and **5b**, 20 mmol) in THF (50 ml) at 0°C and the reaction mixture was stirred (rt, 6 h). The solution was concentrated and the residue was purified by silica gel chromatograph. The related fractions were collected to afford the formamidobenzenes with isomer phenylformimidic acid. To a solution of formamidobenzene with isomer phenylformimidic acid (10 mmol) and triethylamine (30 mmol) in THF (20 ml) was added phosphoryl trichloride (36 mmol) dropwise at 0°C for 1 h. The mixture was extracted by DCM and the organic phase was washed by Na_2_CO_3_ (sat. aq.), brine, and dried (Na_2_SO_4_), filtered and concentrated. The residue was purified by CombiFlash silica gel chromatography. The final substituted isocyanobenzenes (**6a** and **6b**) were afforded.

### Diethyl (4-isocyanophenyl)phosphonate (6a)

Treatment of diethyl (4-aminophenyl)phosphonate (**5a**) as outlined in general procedure A provided the title compound (**6a**) as a yellow oil (89% yield). ^1^H NMR (400 MHz, CDCl_3_) δ 7.82 (ddd, *J* = 13.0, 8.5, 2.3 Hz, 2H), 7.43 (ddd, *J* = 8.4, 3.6, 1.9 Hz, 2H), 4.17–3.98 (m, 4H), 1.29 (td, *J* = 7.0, 2.5 Hz, 6H). ^13^C NMR (101 MHz, CDCl_3_) δ 166.98, 133.04 (d, *J* = 10.3 Hz, 2C), 130.37 (d, *J* = 189.7 Hz, 1C), 129.55, 126.39 (d, *J* = 15.4 Hz, 2C), 62.49 (d, *J* = 5.6 Hz, 2C), 16.29 (d, *J* = 6.3 Hz, 2C). ^31^P NMR (162 MHz, CDCl_3_) δ 15.87. ESI-MS m/z: 240.10 (MH^+^), 479.20 (M_2_H^+^).

### Diethyl (4-isocyanobenzyl)phosphonate (6b)

Treatment of diethyl (4-aminobenzyl)phosphonate (**5b**) as outlined in general procedure A provided the title compound (**6b**) as a light green oil (62% yield). ^1^H NMR (400 MHz, CDCl_3_) δ 7.34 (d, *J* = 2.2 Hz, 4H), 4.04 (dqd, *J* = 8.4, 7.0, 1.3 Hz, 4H), 3.16 (d, *J* = 22.0 Hz, 2H), 1.26 (t, *J* = 7.1 Hz, 6H). ^13^C NMR (101 MHz, CDCl_3_) δ 164.34 (s, 1C), 133.71 (d, *J* = 9.3 Hz, 1C), 130.75 (d, *J* = 6.6 Hz, 2CH), 126.43 (d, *J* = 3.1 Hz, 2CH), 125.30 (s, 1C), 62.25 (d, *J* = 6.8 Hz, 2CH_2_), 33.58 (d, *J* = 138.2 Hz, 1CH_2_), 16.34 (d, *J* = 6.0 Hz, 2CH_3_). ^13^C NMR (101 MHz, CDCl_3_) δ 164.34, 133.71 (d, *J* = 9.3 Hz, 1C), 130.75 (d, *J* = 6.6 Hz, 2C), 126.43 (d, *J* = 3.1 Hz, 2C), 125.30, 62.25 (d, *J* = 6.8 Hz, 2C), 33.58 (d, *J* = 138.2 Hz, 1C), 16.34 (d, *J* = 6.0 Hz, 2C). ^31^P NMR (162 MHz, CDCl_3_) δ 24.83. ESI-MS m/z: 254.20 (MH^+^), 507.20 (M_2_H^+^).

### General procedure B to prepare compounds (9a—9h) via GBBR multicomponent reaction

Pyrazine-2-amnes or pyridine-2-amines (**7a**—**7c**, 1 mmol), aldehydes (**8a** and **8b**, 1 mmol), and acetic acid (3 mmol) were mixed in MeOH (3 ml) (rt, 20 min). Isonitriles (**6a** and **6b**, 1 mmol) was added. The reaction solution was stirred (rt, 18 h). The final reaction mixture was concentrated and purified by CombiFlash using silica gel chromograph with DCM and MeOH as eluent. The related fractions were collected to provide final compounds (**9a**—**9h**).

### Diethyl (4-((2-phenylimidazo [1,2-a]pyrazin-3-yl)amino)phenyl)phosphonate (9a)

Treatment of pyrazin-2-amine (**7a**), benzaldehyde (**8a**) and diethyl (4-isocyanophenyl)phosphonate (**6a**) as outlined in general procedure B provided diethyl (4-((2-phenylimidazo [1,2-a]pyrazin-3-yl)amino)phenyl)phosphonate (**9a**) as a brown oil (44% yield). DUIS-MS m/z: 423(MH^+^), 845(Na^+^); 421(M-H)^-^. ESI-MS m/z: 423.20 (MH^+^), 845.30 (M_2_H^+^).

### Diethyl (4-((2-phenylimidazo [1,2-a]pyridin-3-yl)amino)phenyl)phosphonate (9b)

Treatment of pyridin-2-amine (**7b**), benzaldehyde (**8a**) and diethyl (4-isocyanophenyl)phosphonate (**6a**) as outlined in general procedure B provided the title compound (**9b**) as a brown oil (49% yield). ^1^H NMR (400 MHz, CDCl_3_) δ 7.96–7.92 (m, 2H), 7.74 (d, *J* = 6.7 Hz, 1H), 7.59 (dd, *J* = 8.8, 3.7 Hz, 2H), 7.55 (d, *J* = 8.4 Hz, 1H), 7.34–7.24 (m, 3H), 7.24–7.17 (m, 1H), 6.77–6.72 (m, 2H), 6.59 (dd, *J* = 8.4, 3.4 Hz, 2H), 4.09–3.97 (m, 4H), 1.27 (t, *J* = 7.1 Hz, 6H). ^13^C NMR (101 MHz, CDCl_3_) δ 148.78 (d, *J* = 3.2 Hz, 1C), 142.86, 139.48, 133.85 (d, *J* = 11.3 Hz, 2C), 133.07, 128.58 (2C), 127.98, 127.02 (2C), 125.30, 122.66, 117.66 (d, *J* = 197.3 Hz), 117.65, 116.95, 113.11 (d, *J* = 15.9 Hz, 2C), 112.40, 61.90 (d, *J* = 5.4 Hz, 2C), 16.31 (d, *J* = 6.6 Hz, 2C). ^31^P NMR (162 MHz, CDCl_3_) δ 20.21. ESI-MS m/z: 422.20 (MH^+^), 444.10 (MNa^+^), 843.30 (M_2_H^+^), 865.30 (M_2_Na^+^).

### Diethyl (4-((2,7-diphenylimidazo [1,2-a]pyridin-3-yl)amino)phenyl)phosphonate (9c)

Treatment of 4-phenylpyridin-2-amine (**7c**), benzaldehyde (**8a**) and diethyl (4-isocyanophenyl)phosphonate (**6a**) as outlined in general procedure B provided the title compound (**9c**) as a brown oil (18% yield). ^1^H NMR (400 MHz, CDCl_3_) δ 7.92–7.88 (m, 2H), 7.86 (s, 1H), 7.74 (d, *J* = 7.1 Hz, 1H), 7.60 (d, *J* = 7.8 Hz, 3H), 7.56 (d, *J* = 8.6 Hz, 1H), 7.46 (t, *J* = 7.4 Hz, 2H), 7.42–7.37 (m, 1H), 7.32–7.27 (m, 2H), 7.24 (t, *J* = 7.2 Hz, 1H), 7.00 (dd, *J* = 7.1, 1.7 Hz, 1H), 6.90 (s, 1H), 6.63 (dd, *J* = 8.3, 3.4 Hz, 2H), 4.11–3.96 (m, 4H), 1.27 (t, *J* = 7.1 Hz, 6H). ^13^C NMR (101 MHz, CDCl_3_) δ 148.81 (d, *J* = 3.3 Hz, 1C), 143.02, 139.40, 138.92, 138.23, 133.88 (d, *J* = 11.4 Hz, 2C), 132.34, 129.15 (2C), 128.63 (2C), 128.52, 128.18, 127.04 (2C), 126.80 (2C), 122.62, 117.32 (d, *J* = 198.1 Hz, 1C), 116.92, 113.80, 113.22 (d, *J* = 15.9 Hz, 2C), 112.63, 62.11 (d, *J* = 5.5 Hz, 2C), 16.28 (d, *J* = 6.6 Hz, 2C). ^31^P NMR (162 MHz, CDCl_3_) δ 20.37. ESI-MS m/z: 498.20 (MH^+^).

### Diethyl (4-((2-(2-hydroxyphenyl)imidazo [1,2-a]pyridin-3-yl)amino)phenyl)phosphonate (9d)

Treatment of pyridin-2-amine (**7b**), 2-hydroxybenzaldehyde (**8b**) and diethyl (4-isocyanophenyl)phosphonate (**6a**) as outlined in general procedure B provided diethyl (4-((2-(2-hydroxyphenyl)imidazo [1,2-a]pyridin-3-yl)amino)phenyl)phosphonate (**9d**) as a yellow oil (81% yield). DUIS-MS m/z: 438 (MH^+^), 875 (M_2_H^+^).

### Diethyl (4-((2-(2-hydroxyphenyl)-7-phenylimidazo [1,2-a]pyridin-3-yl)amino)phenyl)phosphonate (9e)

Treatment of 4-phenylpyridin-2-amine (**7c**), 2-hydroxybenzaldehyde (**8b**) and diethyl (4-isocyanophenyl)phosphonate (**6a**) as outlined in general procedure B provided diethyl (4-((2-(2-hydroxyphenyl)-7-phenylimidazo [1,2-a]pyridin-3-yl)amino)phenyl)phosphonate (**9e**) as a brown oil (73% yield). DUIS-MS m/z: 515 (MH^+^), 512 (M-H)^-^.

### Diethyl (4-((2-phenylimidazo [1,2-a]pyrazin-3-yl)amino)benzyl)phosphonate (9f)

Treatment of pyrazin-2-amine (**7a**), benzaldehyde (**8a**) and diethyl (4-isocyanobenzyl)phosphonate (**6b**) as outlined in general procedure B provided the title compound (**9f**) as a yellow oil (33% yield). ^1^H NMR (500 MHz, CDCl_3_) δ 9.11 (d, *J* = 1.5 Hz, 1H), 8.01–7.98 (m, 1H), 7.86 (d, *J* = 4.5 Hz, 1H), 7.76 (dd, *J* = 4.5, 1.5 Hz, 1H), 7.41–7.37 (m, 2H), 7.36–7.32 (m, 1H), 7.17 (dd, *J* = 8.6, 2.6 Hz, 2H), 6.55–6.51 (m, 2H), 5.84 (s, 1H), 5.30 (s, 1H), 4.05–3.97 (m, 4H), 3.07 (d, *J* = 21.1 Hz, 2H), 1.24 (t, *J* = 7.1 Hz, 6H). ^13^C NMR (126 MHz, CDCl_3_) δ 143.64, 142.73 (d, *J* = 3.3 Hz, 1C), 141.57, 137.84, 132.49, 131.30 (d, *J* = 6.6 Hz, 2C), 129.50, 128.81 (2C), 128.70, 127.33 (2C), 123.33 (d, *J* = 9.2 Hz, 1C), 119.67, 115.90, 113.90 (d, *J* = 3.0 Hz, 2C), 62.07 (d, *J* = 6.8 Hz, 2C), 32.73 (d, *J* = 139.1 Hz, 1C), 16.40 (d, *J* = 5.9 Hz, 2C). ^31^P NMR (162 MHz, CDCl_3_) δ 26.77. ESI-MS m/z: 437.20 (MH^+^), 871.3 (M_2_H^+^).

### Diethyl (4-((2-phenylimidazo [1,2-a]pyridin-3-yl)amino)benzyl)phosphonate (9g)

Treatment of pyridin-2-amine (**7b**), benzaldehyde (**8a**) and diethyl (4-isocyanobenzyl)phosphonate (**6b**) as outlined in general procedure B provided the title compound (**9g**) as a colorless oil (46% yield). ^1^H NMR (400 MHz, CDCl_3_) δ 8.00–7.94 (m, 2H), 7.74 (dt, *J* = 6.8, 1.2 Hz, 1H), 7.58 (dt, *J* = 9.0, 1.1 Hz, 1H), 7.34–7.22 (m, 3H), 7.16 (ddd, *J* = 9.1, 6.7, 1.3 Hz, 1H), 7.09 (dd, *J* = 8.5, 2.6 Hz, 2H), 6.69 (td, *J* = 6.7, 1.1 Hz, 1H), 6.50 (d, *J* = 7.8 Hz, 2H), 6.07 (s, 1H), 3.97 (dddd, *J* = 10.5, 8.2, 6.5, 3.2 Hz, 4H), 3.02 (d, *J* = 21.0 Hz, 2H), 1.19 (t, *J* = 7.1 Hz, 6H). ^13^C NMR (101 MHz, CDCl_3_) δ 143.84 (d, *J* = 3.3 Hz, 1C), 142.68, 139.16, 133.41, 131.07 (d, *J* = 6.5 Hz, 2C), 128.51 (2C), 127.76, 127.03 (2C), 124.97, 122.83, 122.21 (d, *J* = 9.2 Hz, 1C), 118.29, 117.55, 113.64 (d, *J* = 2.9 Hz, 2C), 112.07, 62.03 (d, *J* = 6.8 Hz, 2C), 32.68 (d, *J* = 138.9 Hz, 1C), 16.34 (d, *J* = 6.0 Hz, 2C). ^31^P NMR (162 MHz, CDCl_3_) δ 27.00. ESI-MS m/z: 436.20 (MH^+^), 871.40 (M_2_H^+^).

### Diethyl (4-((2,7-diphenylimidazo [1,2-a]pyridin-3-yl)amino)benzyl)phosphonate (9h)

Treatment of 4-phenylpyridin-2-amine (**7c**), benzaldehyde (**8a**) and diethyl (4-isocyanobenzyl)phosphonate (**6b**) as outlined in general procedure B provided diethyl (4-((2,7-diphenylimidazo [1,2-a]pyridin-3-yl)amino)benzyl)phosphonate (**9h**) as a colorless oil (74% yield). ESI-MS m/z: 512.20 (MH^+^).

### General procedure C for deprotection of diethyl phosphonates (9a—9h) to prepare phosphonic acids (3a—3e and 4a—4c)

To a solution of diethyl phosphonates (**9a**—**9h**, 0.2 mmol) in acetonitrile (2 ml) was added bromotrimethylsilane (1.0 mmol). The reaction mixture was stirred (rt, 2h). The reaction was quenched by adding MeOH (2 ml) and the result solution was purified by preparative HPLC over 20 min with a flow rate 20 ml/min. The related fractions were concentrated by lyophilizer, phosphonic acids (**3a**—**3e** and **4a**—**4c**) were afforded.

### (4-((2-Phenylimidazo [1,2-a]pyrazin-3-yl)amino)phenyl)phosphonic acid (3a)

Treatment of diethyl (4-((2-phenylimidazo [1,2-a]pyrazin-3-yl)amino)phenyl)phosphonate (**9a**) as outlined in general procedure C and purification by preparative HPLC (linear gradient of 10% B to 30% B over 20 min with a flow rate 20 ml/min, retention time = 12.0 min) provided the title compound (**3a**) as a yellow solid (53% yield). ^1^H NMR (400 MHz, DMSO-d_6_) δ 9.08 (d, *J* = 1.5 Hz, 1H), 8.70 (s, 1H), 8.00–7.95 (m, 3H), 7.84 (d, *J* = 4.6 Hz, 1H), 7.41 (d, *J* = 8.6 Hz, 1H), 7.37 (t, *J* = 8.0 Hz, 3H), 7.31–7.26 (m, 1H), 6.51 (dd, *J* = 8.2, 3.0 Hz, 2H). ^13^C NMR (126 MHz, DMSO-d6) δ 147.63 (d, *J* = 3.1 Hz, 1C), 143.29, 140.29, 137.71, 133.04, 133.00 (d, *J* = 11.5 Hz, 2C), 129.56, 129.21 (2C), 128.96, 127.19 (2C), 123.91 (d, *J* = 189.4 Hz, 1C), 120.28, 117.04, 113.01 (d, *J* = 15.0 Hz, 2C). ^31^P NMR (162 MHz, DMSO-d_6_) δ 14.09. HRMS calcd. For C_18_H_16_N_4_O_3_P (MH^+^), 367.0955; found, 367.0941.

### (4-((2-Phenylimidazo [1,2-a]pyridin-3-yl)amino)phenyl)phosphonic acid (3b)

Treatment of diethyl (4-((2-phenylimidazo [1,2-a]pyridin-3-yl)amino)phenyl)phosphonate (**9b**) as outlined in general procedure C and purification by preparative HPLC (linear gradient of 10% B to 20% B over 20 min with a flow rate 20 ml/min, retention time = 9.9 min) provided the title compound (**3b**) as a white solid (55% yield). ^1^H NMR (400 MHz, DMSO-d_6_) δ 8.83 (s, 1H), 8.20 (d, *J* = 6.8 Hz, 1H), 8.02–7.98 (m, 2H), 7.88 (d, *J* = 9.0 Hz, 1H), 7.70 (t, *J* = 8.0 Hz, 1H), 7.51 (dt, *J* = 8.6, 6.9 Hz, 4H), 7.46–7.41 (m, 1H), 7.24 (t, *J* = 6.8 Hz, 1H), 6.70 (dd, *J* = 8.3, 3.0 Hz, 2H). ^31^P NMR (162 MHz, DMSO-d_6_) δ 14.00.^13^C NMR (126 MHz, DMSO-d_6_) δ 147.74 (d, *J* = 3.0 Hz, 1C), 140.55, 134.16, 132.96 (d, *J* = 11.1 Hz, 2C), 130.54, 129.88, 129.40 (3C), 127.12 (2C), 124.51, 124.15 (d, *J* = 189.2 Hz, 1C), 119.34, 115.57, 115.29, 113.08 (d, *J* = 15.0 Hz, 2C). DUIS-MS m/z: 366 (MH^+^), 731 (M_2_H^+^). HRMS calcd. For C_19_H_17_N_3_O_3_P (MH^+^), 366.1002; found, 366.1018.

### (4-((2,7-Diphenylimidazo [1,2-a]pyridin-3-yl)amino)phenyl)phosphonic acid (3c)

Treatment of diethyl (4-((2,7-diphenylimidazo [1,2-a]pyridin-3-yl)amino)phenyl)phosphonate (**9c**) as outlined in general procedure C and purification by preparative HPLC (linear gradient of 10% B to 40% B over 20 min with a flow rate 20 ml/min, retention time = 15.9 min) provided the title compound (**3c**) as a white solid (52% yield). ^1^H NMR (400 MHz, DMSO-d_6_) δ 8.74 (s, 1H), 8.10 (d, *J* = 7.1 Hz, 1H), 8.05–8.00 (m, 3H), 7.89–7.85 (m, 2H), 7.58–7.51 (m, 3H), 7.51–7.43 (m, 6H), 7.39–7.34 (m, 1H), 6.66 (dd, *J* = 8.3, 3.1 Hz, 2H). ^13^C NMR (126 MHz, DMSO-d6) δ 148.54 (d, *J* = 3.1 Hz, 1C), 142.39, 139.65, 138.30, 137.31, 133.48 (d, *J* = 11.0 Hz, 2C), 132.72, 130.17 (2C), 129.69 (2C), 129.26, 127.74 (2C), 127.46 (2C), 124.31 (d, *J* = 189.3 Hz, 1C), 124.59, 119.32, 119.13, 116.75, 113.77, 113.43 (d, *J* = 15.5 Hz, 2C). ^31^P NMR (162 MHz, DMSO-d_6_) δ 14.11. HRMS calcd. For C_25_H_21_N_3_O_3_P (MH^+^), 442.1315; found, 442.1332.

### (4-((2-(2-Hydroxyphenyl)imidazo [1,2-a]pyridin-3-yl)amino)phenyl)phosphonic acid (3d)

Treatment of diethyl (4-((2-(2-hydroxyphenyl)imidazo [1,2-a]pyridin-3-yl)amino)phenyl)phosphonate (**9d**) as outlined in general procedure C and purification by preparative HPLC (linear gradient of 5% B to 20% B over 20 min with a flow rate 20 ml/min, retention time = 14.1 min) provided the title compound (**3d**) as a white solid (42% yield). ^1^H NMR (500 MHz, DMSO-d_6_) δ 8.64 (s, 1H), 8.04 (d, *J* = 6.8 Hz, 1H), 7.80–7.75 (m, 1H), 7.75 (d, *J* = 9.0 Hz, 1H), 7.48 (t, *J* = 8.1 Hz, 1H), 7.42 (d, *J* = 8.5 Hz, 1H), 7.39 (d, *J* = 8.5 Hz, 1H), 7.13 (ddd, *J* = 8.6, 7.2, 1.7 Hz, 1H), 7.07 (t, *J* = 6.8 Hz, 1H), 6.88 (dd, *J* = 8.3, 1.3 Hz, 1H), 6.73 (ddd, *J* = 8.1, 7.3, 1.2 Hz, 1H), 6.55 (d, *J* = 6.6 Hz, 2H). ^13^C NMR (126 MHz, DMSO-d_6_) δ 156.85, 147.39 (d, *J* = 3.0 Hz, 1C), 139.50, 135.33, 132.56 (d, *J* = 11.0 Hz, 2C), 129.90, 127.75, 126.87, 123.46 (d, *J* = 189.4 Hz, 1C), 123.26, 119.01, 117.69, 116.94, 115.81, 115.52, 114.19, 112.51 (d, *J* = 15.0 Hz, 2C). ^31^P NMR (162 MHz, DMSO-d6) δ 14.06. DUIS-MS m/z: 382 (MH^+^). HRMS calcd. For C_19_H_17_N_3_O_4_P (MH^+^), 382.0951; found, 382.0940.

### (4-((2-(2-Hydroxyphenyl)-7-phenylimidazo [1,2-a]pyridin-3-yl)amino)phenyl)phosphonic acid (3e)

Treatment of diethyl (4-((2-(2-hydroxyphenyl)-7-phenylimidazo [1,2-a]pyridin-3-yl)amino)phenyl)phosphonate (**9e**) as outlined in general procedure C and purification by preparative HPLC (linear gradient of 10% B to 40% B over 20 min with a flow rate 20 ml/min, retention time = 15.9 min) provided the title compound (**3e**) as a yellow solid (48% yield). ^1^H NMR (500 MHz, DMSO-d6) δ 8.78 (s, 1H), 8.19 (d, *J* = 7.1 Hz, 1H), 8.15 (s, 1H), 7.92–7.87 (m, 2H), 7.83 (d, *J* = 7.7 Hz, 1H), 7.56 (t, *J* = 7.7 Hz, 3H), 7.50 (dd, *J* = 12.5, 8.6 Hz, 3H), 7.27–7.22 (m, 1H), 6.99 (d, *J* = 7.7 Hz, 1H), 6.84 (td, *J* = 7.6, 1.2 Hz, 1H), 6.69 (d, *J* = 5.3 Hz, 2H). ^13^C NMR (126 MHz, DMSO-d_6_) δ 157.63, 148.24 (d, *J* = 3.1 Hz, 1C), 140.56, 137.93, 133.49 (d, *J* = 11.2 Hz, 2C), 131.17, 130.41, 130.23 (2C), 130.00, 128.19, 128.06, 127.87 (2C), 124.56, 124.55 (d, *J* = 189.4 Hz, 1C), 120.04, 118.87, 117.82, 116.07, 114.66, 113.60 (d, *J* = 15.0 Hz, 2C), 112.49.^31^P NMR (162 MHz, DMSO-d6) δ 14.06. HRMS calcd. For C_25_H_21_N_3_O_4_P (MH^+^), 458.1264; found, 458.1249.

### (4-((2-Phenylimidazo [1,2-a]pyrazin-3-yl)amino)benzyl)phosphonic acid (4a)

Treatment of diethyl (4-((2-phenylimidazo [1,2-a]pyrazin-3-yl)amino)benzyl)phosphonate (**9f**) as outlined in general procedure C and purification by preparative HPLC (linear gradient of 10% B to 30% B over 20 min with a flow rate 20 ml/min, retention time = 15.1 min) provided the title compound (**4a**) as an orange solid (57% yield). ^1^H NMR (500 MHz, DMSO-d_6_) δ 9.18 (d, *J* = 1.4 Hz, 1H), 8.38 (s, 1H), 8.12 (dd, *J* = 8.4, 1.3 Hz, 2H), 8.04 (dd, *J* = 4.6, 1.5 Hz, 1H), 7.94 (d, *J* = 4.6 Hz, 1H), 7.48 (t, *J* = 7.6 Hz, 2H), 7.41–7.36 (m, 1H), 7.07 (dd, *J* = 8.7, 2.4 Hz, 2H), 6.49 (d, *J* = 8.1 Hz, 2H), 2.84 (d, *J* = 20.9 Hz, 2H). ^13^C NMR (126 MHz, DMSO-d_6_) δ 143.32 (d, *J* = 2.9 Hz, 1C), 143.01, 140.27, 137.45, 133.14, 131.33 (d, *J* = 6.2 Hz, 2C), 129.19, 129.16 (2C), 128.87, 127.24 (2C), 124.94 (d, *J* = 8.8 Hz, 1C), 121.53, 117.08, 113.44 (d, *J* = 2.7 Hz, 2C), 34.86 (d, *J* = 133.4 Hz). ^31^P NMR (162 MHz, DMSO-d_6_) δ 21.68. HRMS calcd. For C_19_H_18_N_4_O_3_P (MH^+^), 381.1111; found, 381.1100.

### (4-((2-Phenylimidazo [1,2-a]pyridin-3-yl)amino)benzyl)phosphonic acid (4b)

Treatment of diethyl (4-((2-phenylimidazo [1,2-a]pyridin-3-yl)amino)benzyl)phosphonate (**9g**) as outlined in general procedure C and purification by preparative HPLC (linear gradient of 10% B to 40% B over 20 min with a flow rate 20 ml/min, retention time = 11.0 min) provided the title compound (**4b**) as a white solid (67% yield). ^1^H NMR (400 MHz, DMSO-d_6_) δ 8.25 (s, 1H), 8.04 (d, *J* = 6.8 Hz, 1H), 7.95–7.90 (m, 2H), 7.73 (d, *J* = 9.0 Hz, 1H), 7.54 (t, *J* = 8.0 Hz, 1H), 7.40 (t, *J* = 7.6 Hz, 2H), 7.34–7.27 (m, 1H), 7.09 (t, *J* = 6.8 Hz, 1H), 6.97 (dd, *J* = 8.7, 2.4 Hz, 2H), 6.45 (d, *J* = 8.1 Hz, 2H), 2.74 (d, *J* = 20.9 Hz, 2H). ^13^C NMR (126 MHz, DMSO-d_6_) δ 143.51 (d, *J* = 2.9 Hz, 1C), 140.50, 134.21, 131.32 (d, *J* = 6.3 Hz, 2C), 130.98, 129.31 (3C), 129.14, 127.11 (2C), 125.04 (d, *J* = 8.8 Hz, 1C), 124.42, 120.32, 115.66, 114.93, 113.42 (d, *J* = 2.6 Hz, 2C), 34.86 (d, *J* = 133.2 Hz, 1C). ^31^P NMR (162 MHz, DMSO-d_6_) δ 21.64. DUIS-MS m/z: 380 (MH^+^), 759 (M_2_H^+^). HRMS calcd. For C_20_H_19_N_3_O_3_P (MH^+^), 380.1159; found, 380.1176.

### (4-((2,7-Diphenylimidazo [1,2-a]pyridin-3-yl)amino)benzyl)phosphonic acid (4c)

Treatment of diethyl (4-((2,7-diphenylimidazo [1,2-a]pyridin-3-yl)amino)benzyl)phosphonate (**9h**) as outlined in general procedure C and purification by preparative HPLC (linear gradient of 10% B to 40% B over 20 min with a flow rate 20 ml/min, retention time = 17.2 min) provided the title compound (**4c**) as a white solid (84% yield). ^1^H NMR (500 MHz, DMSO-d_6_) δ 8.42 (s, 1H), 8.20 (d, *J* = 7.1 Hz, 1H), 8.09 (s, 1H), 8.06 (d, *J* = 7.3 Hz, 2H), 7.92 (d, *J* = 7.4 Hz, 2H), 7.61 (dd, *J* = 8.4, 6.8 Hz, 3H), 7.53 (td, *J* = 7.6, 2.8 Hz, 3H), 7.44 (t, *J* = 7.5 Hz, 1H), 7.10 (dd, *J* = 8.6, 2.4 Hz, 2H), 6.61 (d, *J* = 8.1 Hz, 2H), 2.87 (d, *J* = 20.9 Hz, 2H). ^13^C NMR (126 MHz, DMSO-d_6_) δ 143.51 (d, *J* = 3.1 Hz, 1C), 140.92 (2C), 138.82, 137.47, 131.36 (d, *J* = 6.1 Hz, 2C), 130.94, 129.78 (2C), 129.58, 129.34 (2C), 129.21, 127.43 (2C), 127.12 (2C), 125.11 (d, *J* = 8.6 Hz, 1C), 124.57, 120.27, 114.13, 113.50 (d, *J* = 2.7 Hz, 2C), 111.74, 34.88 (d, *J* = 133.2 Hz, 1C). ^31^P NMR (162 MHz, DMSO-d_6_) δ 21.66. DUIS-MS m/z: 456 (MH^+^), 911(M_2_H^+^). HRMS calcd. For C_26_H_23_N_3_O_3_P(MH^+^), 456.1472; found, 456.1478.

### *In vitro* TDP1 gel-based assays

The inhibition of TDP1 was also conducted according to gel-based as previously described (Lountos et al., 2019; Zhao et al., 2021). Briefly, the DNA substrate (1 nM, 5′Cy5-GATCTAAAAGACTT-pY-3′) was incubated with recombinant full-length TDP1 or TDP1 (148–608) (40 p.m.) in the absence or presence of inhibitors for 15 min at room temperature in TDP1 reaction buffer (50 mM Tris HCl, pH 7.5, 80 mM KCl, 2 mM EDTA, 1 mM DTT, 40 μg/ml BSA and 0.01% Tween-20). Reactions were stopped by adding an equal volume of gel loading buffer (99.5% (v/v) formamide, 5 mM EDTA). Samples were then subjected to a 20% denaturing PAGE gel following by gel scanning using a Typhoon FLA 9500 scanner (GE Healthcare). The IC_50_ values of TDP1 inhibitors were calculated by comparing the percentage of cleavage product (5′Cy5-GATCTAAAAGACTT-p-3′) to DMSO control.

### X-ray crystallography

The catalytic domain of TDP1 (consisting of residues S148-S608) was expressed and purified for crystallographic studies as previously reported (Lountos et al., 2019). Crystals were grown by the hanging drop vapor diffusion method by mixing 2 μL of TDP1 (22 mg/ml in 25 mM Tris-HCL pH 7.2, 150 mM sodium chloride, and 2 mM tris(2-carboxyethyl)phosphine buffer) with 2 μL of well solution (0.1M MOPS/HEPES-Na, pH 7.5, 10% (w/v) PEG 8000, 20% (v/v) ethylene glycol, 0.03 M sodium fluoride, 0.03 M sodium bromide, 0.03 M sodium iodide) and sealed over 500 μL of well solution in a Nextal 15-well crystallization plate. The size of the crystals was subsequently improved with streak-seeding. For soaking experiments, stock solutions of **4c** (124.3 mM) and **3b** (177.6 mM) were prepared in 100% DMSO. Crystals of TDP1 were then transferred to a 4 μL drop solution consisting of well solution supplemented with either 12.4 mM **4c** or 17.8 mM **3b** and a final concentration of 10% (v/v) DMSO. The drops were then sealed over 500 μL of well solution and the crystals were soaked for 5 days. Crystals for data collection were retrieved from the drops using a litholoop and immediately flash-cooled by plunging into liquid nitrogen without the need of additional cryoprotectant.

X-ray diffraction data were collected remotely at beamline 22-BM of the SER-CAT facility, Advanced Photon Source, Argonne National Laboratory. For both data sets, X-ray diffraction data were collected with a Rayonix MX300-HS detector using an X-ray wavelength of 1.0000 Å, an oscillation angle of 1.0°, an exposure time of 4 s, and a crystal to detector distance of 200 mm. The X-ray data sets were processed using HKL3000 (Minor et al., 2006). The structures were solved by molecular replacement using the coordinates of a previous structure of TDP1 (PDB code: 6DHU) (Lountos et al., 2019) with all solvent and ligand atoms deleted and the program PHASER (McCoy et al., 2007) in the Phenix crystallographic software suite (Liebschner et al., 2019). The resulting electron density maps were examined for difference electron density features (contoured at 3.0 σ level) to identity the location of the inhibitors. Coordinates for the **4c** and **3b** molecules were prepared using the Molinspiration server (www.molinspiration.com) and the appropriate.cif files for use in refinements were prepared using the eLBOW (Moriarty et al., 2009) feature in Phenix. Iterative rounds of model adjustments and corrections were carried out in Coot (Emsley et al., 2010) followed by refinement in phenix.refine (Afonine et al., 2012). Water molecules were located automatically using Coot and phenix.refine, visually inspected, and analyzed with UnDowser (Prisant et al., 2020) in MolProbity (Chen et al., 2010). Final model quality and validation were performed using MolProbity. Crystallographic data collection and refinement statistics are presented in Supplementary Table S1. The PDB coordinates and structure factors for the structures of TDP1 bound to **4c** and **3b** were deposited into the Protein Data Bank under accession codes 7UFY and 7UFZ, respectively.

## Data Availability

The original contributions presented in the study are publicly available. This data can be found here: https://www.rcsb.org/, 7UFY, 7UFZ.
