# Supplementary material for: Phosphonic acid-containing inhibitors of tyrosyl-DNA phosphodiesterase 1
Source: Front Chem. 2022 Aug 16;10:910953. doi: 10.3389/fchem.2022.910953 (PMC9424690; doi:10.3389/fchem.2022.910953)
Supplement: Supplementary file 1 [file Table1.pdf]

SUPPLEMENTARY MATERIAL

Phosphonic Acid-containing Inhibitors of Tyrosyl-DNA Phosphodiesterase 1

Xue Zhi Zhao[1†*], Wenjie Wang[2†], George T. Lountos[3], Joseph E. Tropea[4], Danielle Needle[4], Yves Pommier[2] and Terrence R. Burke, Jr.[1]

[1]Chemical Biology Laboratory, Center for Cancer Research, National Cancer Institute, National Institutes of Health, Frederick, MD, U.S.A. [2]Developmental Therapeutics Branch & Laboratory of Molecular Pharmacology, Center for Cancer Research, National Cancer Institute, National Institutes of Health, Bethesda, MD, U.S.A. [3]Basic Science Program, Frederick National Laboratory for Cancer Research, Frederick, MD, U.S.A. [4]Center for Structural Biology, Center for Cancer Research, National Cancer Institute, Frederick, MD, U.S.A.

†Co-first authors

Correspondence*:

Xue Zhi Zhao

xuezhi.zhao@nih.gov

Table S1. X-ray Data Collection and Refinement Statistics.

	**TDP1-4c complex**	**TDP1-3b complex**
Data collection Statistics		
Diffraction source	APS, SER-CAT, 22-BM	APS, SER-CAT, 22-BM
Wavelength (Å)	1.0000	1.0000
Temperature (K)	100	100
Detector	MX300-HS	MX300-HS
Space group	$P2_12_12_1$	$P2_12_12_1$
Unit cell parameters		
a=,b=,c= (Å)	49.81, 104.73, 193.15	49.89, 104.60, 193.10
α=,β=,γ= (°)	90, 90, 90	90, 90, 90
Resolution range (Å)	50-1.58 (1.62-1.58)*	50-1.56 (1.59-1.56)
Total reflections	994965	965807
Unique reflections	137386 (6750)	142916 (6267)
Completeness (%)	99.9 (99.6)	98.8 (87.6)
Multiplicity	7.2 (5.6)	6.8 (4.7)
Mean $I/\sigma(I)$	35.8 (2.0)	30.2 (1.8)
R_{merge}	0.068 (0.671)	0.072 (0.652)
$R_{p.i.m.}$	0.027 (0.304)	0.030 (0.286)
$CC_{1/2}$	0.996 (0.848)	0.997 (0.775)
Refinement Statistics		
Resolution range (Å)	49.27-1.58	43.83-1.56
Number of reflections	137247	142797
Number of reflections used in R_{free}	6803	6955
Final R_{work}	0.166	0.171
Final R_{free}	0.193	0.195
Number of non-H atoms		
Protein, chain A	3647	3664
Protein, chain B	3646	3627
4c (XZ766)	66	
3b (XZ768)		74
Water	808	777
Ethylene glycol	28	28
DMSO		4
Average B factors (Å^2)		
Protein, chain A	25.5	22.1
Protein, chain B	32.2	33.1
4c (XZ766)	45.8	
3b (XZ768)		35.5
Water	42.6	38.9
Ethylene glycol	35.4	28.6
DMSO		55.4
Estimated coordinate error (Å)		0.16

R.m.s. deviations from ideal		
Bond lengths (Å)	0.01	0.01
Bond angles (°)	1.1	1.0
Ramachandran plot		
Favored (%)	98.0	97.1
Allowed (%)	2.0	2.8
Outliers (%)	0	0.1
MolProbity Analysis		
Clashscore, all atoms	2.05 (99th percentile)	2.54 (99th percentile)
Protein geometry score	0.97 (100th percentile)	1.20 (98th percentile)
PDB deposition code	7UFY	7UFZ
*Values in parentheses are for the highest resolution shell of data.		
